# The Prognosis of Valvular Heart Disease

**Published:** 1908-02-29

**Authors:** 


### Diseases of Children.

### THE PROGNOSIS OF VALVULAR HEART DISEASE.

The prognosis of heart disease is not now regarded so pessimistically as in the early days during which the use of the stethoscope was becoming general. In children the congenital or acquired character of the lesion has to be considered, as well as numerous other factors. Put generally, the prognosis depends on the establishment and maintenance of compensatory hypertrophy.

### Examination before History.

As a preliminary piece of advice, always learn the exact physical signs and symptoms presented by the heart and other organs before inquiring into the personal and family history. In this way the liability to bias in the interpretation of the results is much diminished. The extent and kind of the lesion is indicated by the number of valves affected; the condition of the myocardium; the presence or absence of pericardial adhesions, and of pain; and the relations of the heart to the neighbouring parts.

In order to estimate the magnitude of the lesion, that is, the amount of injury sustained by the affected part, we must take into consideration the physical signs proper to the lesion. Mere loudness of the murmur is of only secondary importance. Hypertrophy, with or without dilatation, general or partial, is a further guide; but this is determined by the date of the mischief as well as its magnitude.

### Local and Constitutional Changes.

In addition, we must estimate the interference with the proper cardiac circulation, leading to degeneration of the muscle and its results; with the systematic and pulmonary circulations, arterial and venous, leading to changes in nutrition and function of distant organs, such as the brain, liver, spleen, kidneys; and the presence of general or local dropsy.

When actually present, these afford a measure of the degree in which the injury is felt at the time the patient is under observation; as far as the heart is concerned they may be primary or secondary affections. In children they are usually secondary. Thus, fatty degeneration results sometimes from valvular disease, whereas in adults it may be the cause. The presence of hypertrophy is proof that compensation has been more or less completely attained. If the hypertrophy is very great, it is not likely that compensation will be lasting, for the more a muscle exceeds its normal bulk, the more difficult it is to maintain its normal nutrition and functional activity. Dilatation, if it has existed for some time and does not improve at once under treatment, is a proof that compensation has broken down, and may not be regained. From a study of these conditions we are able to estimate the tendency to syncope, pulmonary congestion, embolism, or angina. There are two sets of cases in which the prognosis is especially bad. A temperature persistently hectic in type, sometimes irregular, and an enlarged spleen indicate infective endocarditis. Frequent and severe attacks of cardiac pain are also of serious import.

Passing from the heart, though in association therewith, we must think of the lesion in relation to the constitutional state. Is the lesion traumatic? *i.e.* due to some injury, strain, or acute disease, which sooner or later comes to an end. If so, the primary lesion ceases as an active process, although the mischief already done remains. In children rheumatic fever is the best instance of this, for the valves rarely give way as the result of sudden, violent, momentary effort, or from long continued excessive muscular exertion as in adults.

### Congenital Lesions.

In these and similar cases, if the kind, extent, and the magnitude of the lesion be not very serious, still more if it be wholly or in part congenital, compensation is often all but, or quite, complete, and the patient may enjoy a long and active life. Some varieties of congenital valvular lesions, such as mild stenosis, anomalies in the number of valves, are quite compatible with the normal duration and activities of life. In some cases not only does compensation take place, but the lesion itself is repaired and the obstacle to the blood flow is removed. There is then no need for further propelling power, that is, hypertrophy, and the heart will regain its normal bulk. Few instances of such complete recovery can be proved to have taken place, and the number is far too small to justify a confident prognosis of such recovery in any particular case. Under favourable conditions, especially in rheumatic fever, more or less improvement is so frequent that a comparatively favourable prognosis as regards length of life and power for work may be honestly given. The murmur in rheumatic fever is often partly due to dilatation, which completely disappears.

In adults the cardiac mischief may be part of a general diathetic state leading to degenerative changes. In children this is not the case, but sometimes the effects on the circulation and nutrition are so great that a general cachexia, secondary to the lesion, is produced, and compensation is rendered impossible. General improvement in nutrition and gain in weight are good signs.

### Personal and Social Factors.

Seeing that compensation must be maintained as well as produced, the prognosis further depends on whether the personal and social conditions of the patient are or are not favourable to healthy cardiac nutrition. Youth is favourable, for the nutritive powers are vigorous; so, too, a family predisposition to prolonged life, equable temper and strong will, and the absence of any tendency to disease, general or local, which may affect the heart. The occupation and mode of life should be such that the patient can regulate at will the amount of work and rest. He should be able to obtain changes of climate and scenery needed, a good diet, and freedom from worry and anxiety. Out-of-door exercise is of the utmost importance, as long as it does not cause pain, palpitation, dyspnœa, anorexia, or sleeplessness. These children must not be brought up as chronic invalids.

